# Perceived Anxiety, Coping, and Autonomic Function in Takotsubo Syndrome Long after the Acute Event

**DOI:** 10.3390/life12091376

**Published:** 2022-09-03

**Authors:** Davide Lazzeroni, Chiara Ciraci, Marinella Sommaruga, Carlotta Oggioni, Melissa Saccò, Valentina Ziveri, Letizia Paglialonga, Matteo Bini, Luca Moderato, Lorenzo Brambilla, Paolo Coruzzi, Gianluca Cruciani, Vittorio Lingiardi, Annalisa Tanzilli, Federica Galli

**Affiliations:** 1 IRCCS Fondazione Don C. Gnocchi, 20132 Milan, Italy; 2 Psychology Unit, Istituti Clinici Scientifici Maugeri, IRCSS Milano, 20138 Milano, Italy; 3 Guglielmo da Saliceto Hospital, 29121 Piacenza, Italy; 4 Department of Dynamic and Clinical Psychology, and Health Studies, Sapienza University of Rome, 00185 Rome, Italy

**Keywords:** Takotsubo syndrome, autonomic function, heart rate variability (HRV), coping, anxiety, stress

## Abstract

Background: Anxiety and depressive disorders represent predisposing factors for the autonomic dysfunctions that characterize the acute phase of Takotsubo syndrome (TS). However, there is insufficient data on this relationship after the acute event. The present study aimed at evaluating the psychological and autonomic status of patients with a history of TS. Methods: Ten TS patients whose acute event occurred at least 1 year prior to the evaluation and nine healthy age- and sex-matched subjects were evaluated. The cardiovascular assessment included a clinical examination, beat-to-beat heart rate monitoring to assess heart rate variability, and a psychological examination using the 16 Personality Factors-C Form (16PF), the Acceptance and Action Questionnaire-II, the Coping Orientations to Problems Experienced (COPE), the Beck Depression Inventory-II, and the State-Trait Anxiety Inventory (STAI). Results: TS patients scored significantly higher on the STAI (i.e., Anxiety Trait), 16PF (i.e., Tension), and COPE (i.e., Transcendental Orientation). TS patients also showed lower heart rate variability. Moreover, a significant inverse correlation was found between sympathetic tone (LF/HF ratio) and coping orientation. Conclusions: Long after the acute event, TS patients are characterized by elevated anxiety, high tension, and a specific religious coping strategy.

## 1. Introduction

The American Heart Association [1] recognizes the pivotal role played by psychosomatic factors in the development of cardiovascular disorders. However, the psychophysiological mechanisms and pathways that connect the mind and the heart are not yet clear and deserve wide consideration by the scientific community.

Takotsubo syndrome (TS) is an acute coronary syndrome (ACS) characterized by severe left ventricular (LV) dysfunction that typically recovers spontaneously, within days or weeks, in the absence of obstructive coronary artery disease [2]. It predominantly affects postmenopausal women, and as many as 80% of patients report a precipitant major identifiable stressful event [3]. The acute presentation of TS is usually characterized by symptoms, including dyspnea, angina, palpitations, or syncope, as a consequence of acute LV systolic dysfunction (apical ballooning), acute LV diastolic dysfunction, mitral regurgitation, LV outflow tract obstruction, or acute right ventricular involvement, associated with electrocardiogram (ECG) abnormalities and elevation in cardiac biomarkers that mimic an ACS [4]. Several pathophysiological mechanisms for TS have been proposed, including multivessel coronary vasospasm, abnormal coronary microvascular function, and catecholamine-mediated cardiotoxicity; however, the etiology and pathophysiology of TS are not yet clear [5,6].

While psychological risk factors (i.e., anxiety/mood disorders, chronic stress, personality traits) have been progressively recognized as significant predisposing factors [7,8,9,10], the relationship between TS and the pathophysiologic milieu is not well understood. For example, depression has been proposed as a risk factor for cardiovascular disease, and depressive episodes have been observed in 15–20% of cardiopathic patients [11]. Moreover, it has been reported that, following the acute phase of a cardiac event, 50% of patients with diagnosed major depression experience chronic depression or relapse, with 40% of patients with minor depressive symptoms developing a major depressive episode within 1 year [12,13]. Similarly, anxiety disorders are considered both risk factors [14] and outcomes of cardiac events, with 20–30% of patients reporting an increase in anxiety following a cardiac episode and 50% of this subset reporting chronic anxiety [15].

Coping strategies seem pivotal in the relationship between stress management and unfavorable cardiac outcomes; for instance, Hefner and colleagues showed that TS patients used positive coping strategies (e.g., de-emphasis, positive self-instruction) significantly less frequently than healthy controls, suggesting that the pathogenesis of cardiac episodes may be sensitive to not only an individual’s number of stressors, but also their manner of coping with stressors [16]. Finally, personality traits may affect the onset of cardiovascular disease, though the relationship between personality and TS remains unclear. A recent systematic review showed inconsistent data, with half of the reviewed studies reporting no differences between TS patients and healthy controls, and the remaining studies pointing to greater emotionality and a higher prevalence of the Distressed personality (Type-D) in TS patients, compared to controls with myocardial infarction [9].

Impaired sympathovagal balance has been widely demonstrated during the acute phase of TS [17], whereas lower vagal control—in terms of abnormal heart rate variability (HRV)—has been observed in the period to follow [18]. Lower variability in HRV may reflect autonomous nervous system inflexibility, which has been considered indicative of unfavorable health outcomes [19]. Furthermore, HRV has also been identified as a marker of psychopathology, with lower HRV levels traceable in several psychiatric disorders [20].

Accordingly, the present study aimed at evaluating the psychological and autonomic status of TS patients, to provide evidence of potential predisposing psychosomatic factors. More specifically, the study explored potential differences in psychological constructs and HRV parameters between patients and healthy controls. Based on the literature, it was hypothesized that patients would show higher dysfunctional psychopathological traits in terms of anxiety, depression, and coping strategies relative to controls; however, given the lack of published data, no direct hypotheses were drawn regarding the relationship between TS and personality traits. Concerning HRV, it was hypothesized that patients would present more impaired indices of cardiac adaptation with respect to controls.

## 2. Materials and Methods

The experimental group was composed of 10 TS patients (90% women, mean age 71.7 ± 7.1 years) who had experienced their first episode of TS at least 1 year prior to the evaluation. The higher female presence in the experimental group reflects the higher incidence of TS among females in the general population and is aligned with epidemiological studies reporting a higher prevalence of TS in women relative to men [4,6,21]. Nine healthy age- and sex-matched subjects were recruited as a control group (87% female, mean age 72 ± 7 years). TS was diagnosed according to the Mayo Clinic group diagnostic criteria [2,22,23]. Healthy subjects were selected according to the following criteria: the absence of a previous history of cardiovascular disease or the absence of a previous history of chronic kidney or liver disease, which is central to peripheral nervous system diseases. Moreover, controls were pre-screened with ECG and echocardiography in order to exclude, at enrolment, any cardiovascular diseases, especially LV disfunction. Prior to data collection, all subjects provided informed consent to participate. At enrollment (i.e., 12–24 months following the acute TS episode), participants underwent a cardiovascular examination (1 h), followed by a psychological examination (1.5 h). The study was conducted in accordance with the Declaration of Helsinki, and the protocol was approved by the local Ethics Committee of the “Fondazione Don Gnocchi, Milano, Italy”.

### 2.1. Psychological Assessment

The following self-report psychological tests were administered 1 year after patients’ acute episode of TS:

- *16 Personality Factors (16PF)-C Form* ([24]; *italian version* [25]): The 16PF measures 16 independent personality characteristics. The Italian version comprises three forms. The present study used Form C, which consists of 16 subscales: Schizothymic–Cyclothymic, Dull–Bright, Low–High Ego Strength, Submissive–Dominant, Desurgency–Surgency, Low–High Super Ego Strength, Cautious–Bold, Hard–Sensitive, Trusting–Suspicious, Conforming–Eccentric, Naive–Shrewd, Confident–Guilt Prone, Conservative–Radical, Group Adherent–Self-Sufficient, Weak-Willed–Self Disciplined, and Low–High Ergic Tension. Each subscale has six items, except for the Dull–Bright subscale, which has eight items. On Form C, respondents rate 105 statements using three or more response options (depending on the subscale), such as: “Yes”, “I don’t know”, and “No”. Scores for each subscale range from 1–10, and subscales can be combined to assess five broad factors of general personality. Cronbach’s alphas of the different scales in previous studies in the Italian context ranged from 0.75 to 0.95 [26].
- *Acceptance and Action Questionnaire-II (AAQ-II* [27]; *Italian version* [28]): The AAQ–II is a seven-item self-report measure of psychological flexibility and the capacity to get in touch with one’s emotions and act effectively in specific situations. Items are evaluated on a 7-point Likert scale ranging from 1 (*never true*) to 7 (*always true*). Sample items include “I am afraid of my feelings” and “Emotions cause problems in my life”. For the Italian version of the AAQ-II, internal consistency was high (0.83), and test–retest reliability over a 12-month period was modest (0.61).
- *Coping Orientations to Problems Experienced (COPE* [29]; *Italian version* [30]): The COPE self-report measure consists of 60 items that assess 15 coping strategies. Items are rated with respect to four response options, including “Usually I do not” and “I do almost always,” and refer to five dimensions of coping: Social Support, Avoidance Strategies, Positive Attitude, Problem Orientation, and Transcendent Orientation. Cronbach’s alpha reliabilities for the Italian version ranged between 0.43 and 0.93.
- *Beck Depression Inventory-II (BDI-II* [31]; *Italian version* [32]): The BDI-II is widely used to evaluate the presence and intensity of depressive symptoms (according to DSM-IV criteria) over the past 15 days. The questionnaire consists of 21 items with four response options, ranging from 0 (*not present*) to 3 (*severe*). Scores place respondents in one of four categories of symptoms: 0–13 (minimal depression), 14–19 (mild depression), 20–28 (moderate depression), and 29–63 (severe depression). The Italian version of the BDI-II has been shown to have a one-factor structure, adequate internal consistency (αs in the range 0.80–0.87), and test–retest reliability (r = 0.76).
- *State Trait Anxiety Inventory (STAI) Y1 and Y2* ([33]; *Italian version* [34]): The STAI-Y is widely used to measure anxiety. It is composed of 20 items that assess trait anxiety (e.g., “I worry too much over something that really doesn’t matter”) and 20 that assess state anxiety (e.g., “I am tense”, “I am worried”, “I feel calm”). All items are rated on a 4-point scale, with higher scores indicating more severe anxiety symptoms. The Italian version of STAI-Y has shown good psychometric properties, including adequate internal consistency (Cronbach’s α scores = 0.86–0.95), and test–retest reliability (r = 0.31–0.86).

At enrollment, all patients underwent a first interview to evaluate the presence of acute (i.e., within the last month) and/or chronic stressful events.

### 2.2. Cardiovascular Assessment

Cardiovascular assessment involved a clinical examination by means of a 12-lead ECG and beat-to-beat heart rate monitoring (i.e., 10 min in a supine position and 10 min in a sitting position) to assess HRV, which is a well-known marker of autonomic function. Autonomic evaluation took place in a softly lit room free of stimuli and noise. After the NEXFIN^®^ ECG electrodes were placed at a precordial level to detect the tachogram, a 10-min resting ECG recording was collected so that 5 min of the tachogram free from artifacts could be analyzed. The tachogram was carefully reviewed by an expert operator to remove artifacts and extrasystoles. HRV analysis as well as beat-to-beat ECG artifacts corrections were performed using Editing 32 software. Only the traceable tracks with the clearest tachogram were used. All patients underwent at least 3 days of beta-blocker wash-out and 12 h of sympathomimetic substance (e.g., nicotine, caffeine, tea, chocolate) wash-out. HRV time domain, frequency domain, and fractal analysis were conducted [35].

*Time domain analysis*: The time domain represents the time period between successive heartbeats, considering the SDNN, pNN50, and RMSSD. The standard deviation of the inter-beat interval (IBI) of normal sinus beats (SDNN), measured in milliseconds (ms), represents the first marker of HRV evaluation. Both sympathetic nervous system (SNS) and parasympathetic nervous system (PNS) activity contribute to the SDNN [18]: the percentage of adjacent NN intervals that differ from each other by more than 50 ms (pNN50) is closely correlated with PNS activity [35]. The root mean square of successive differences between normal heartbeats (RMSSD) is obtained by calculating each successive time difference between heartbeats (in ms). This reflects the beat-to-beat variance in heart rate. The RMSSD is less susceptible to respiratory influence [36,37] and represents the primary time-domain measure used to estimate the vagally mediated changes reflected in HRV [36].

*Frequency domain analysis*: Frequency domain analysis estimates the distribution of absolute or relative power into four frequency bands. The Task Force of the European Society of Cardiology and the North American Society of Pacing and Electrophysiology divided heart rate oscillations into ultra-low-frequency (ULF), very-low-frequency (VLF), low-frequency (LF), and high-frequency (HF) bands (10). Since the ULF band (≤0.003 Hz) requires a recording period of at least 24 h, ULF was not collected in the present study. The VLF band (0.0033–0.04 Hz) is strongly correlated with the SDNN time-domain measure. Within a typical 5-min sample, there are approximately 0–12 complete periods of oscillation; the physiological mechanisms responsible for activity within this band are not clearly understood [38]. The LF band (0.04–0.15 Hz) was previously referred to as the baroreceptor range, because it mainly reflects baroreceptor activity during resting conditions [38]. LF power may be produced by both the PNS and the SNS. Finally, the HF or respiratory band (0.15–0.40 Hz) reflects parasympathetic activity. Under controlled conditions with normal rates of breathing, HF power can be used to estimate vagal tone [38]. The LF/HF ratio has been used as an index of sympathovagal balance; according to a simplistic view, LF and HF are indices of sympathetic and parasympathetic activity, respectively. Therefore, a high LH/HF ratio is indicative of hyper-adrenergic tone [38].

*Non-linear analysis*: Non-linearity indicates that a relationship between variables cannot be plotted as a straight line. Non-linear measurements index the unpredictability of a time series, which, in the present study, resulted from the complexity of the mechanisms that regulate HRV [38]. Sample entropy (SampEn), which measures the regularity and complexity of a time series, is correlated with vagal tone. Fractal dimension using detrended fluctuation analysis extracts the correlations between successive RR intervals over different time scales and describes the self-similarity properties of RR fluctuations, thus reflecting the baroreceptor reflex [38].

### 2.3. Statistical Analysis

Continuous variables were expressed as means (*M*) and standard deviations (*SD*), while categorical variables were expressed as numbers (*N*) and percentages (%). To compare continuous variables between groups, considering the small number of cases, the Kruskal–Wallis test (i.e., a non-parametric method for independent samples) was used. The Mann–Whitney–Wilcoxon rank-sum test was used to compare group differences with respect to the categorical variables. To analyze possible associations between participants’ autonomic (i.e., cardiovascular evaluation) and psychological status, correlational analyses were run between HRV outcomes and scores on the administered psychological measures. Significance was defined as a *p*-value < 0.05. All statistical analyses were performed using SPSS version 23 (IBM Corporation, Armonk, NY, USA).

## 3. Results

The TS patient and healthy control groups showed no differences in terms of age, sex, family history of cardiovascular disease, dyslipidemia, smoking, diabetes, arterial hypertension, and anti-hypertensive drugs (Table 1).

### 3.1. Psychological Assessment

**Figure 1:**
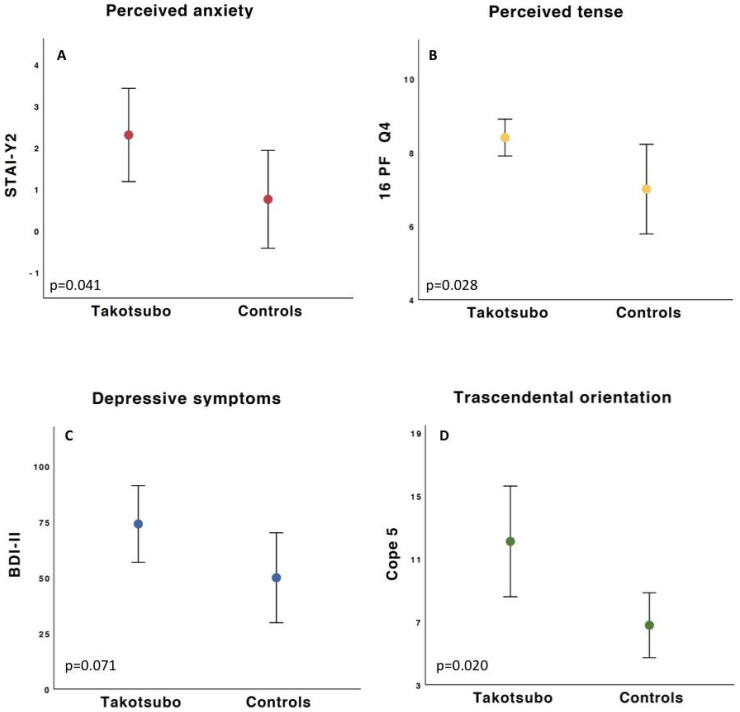
Differences between groups in STAI-Y2 (**A**), 16PF-C Q4 scale (**B**), BDI-II (**C**), and COPE 5 (**D**) scores. Data are shown as mean and standard deviation.

TS was associated with an acute stressful event in six patients (60%), chronic stress in three patients (30%), and no previous stress in only one (10%) patient. Table 2 presents the complete list of stressful events. Within the control sample, only two participants (22%) reported an experience of extreme stress in the past year. The TS group scored significantly higher on the STAI-Y2 (i.e., the Anxiety trait) (*p* = 0.041; see Figure 1A and Table 3) compared to the control group. Additionally, a significant difference on the Cattel scale Q4 (i.e., Tension) (*p* = 0.028) was found in TS patients compared to healthy controls (see Figure 1B and Table 3). Moreover, the 16PF associating variables O+ Q4+ C− and O+ Q4+ C− L− were higher in TS patients with respect to controls (*p* = 0.048 and *p* = 0.047, respectively), suggesting a condition characterized by high Apprehension (O) and Tension (Q4), in association with low Vigilance (L) and Emotional Stability (C). The COPE yielded a significant group difference only on Scale 2 (i.e., Transcendental Orientation), with TS patients registering higher scores (*p* = 0.002; see Figure 1D and Table 3). Concerning the AAQ-II, the TS group was characterized by lower psychological flexibility and a lower capacity to be in contact with their emotions; however, the differences between groups did not reach statistical significance (*p* = 0.072). Additionally, the BDI-II revealed a similar tendency in the total score for Depression (*p* = 0.071; see Figure 1C and Table 3). Table 3 presents an extended description of the psychological test scores and statistical group comparisons.

### 3.2. Autonomic Status (Cardiovascular Evaluation) and Association with Psychological Status

**Figure 2:**
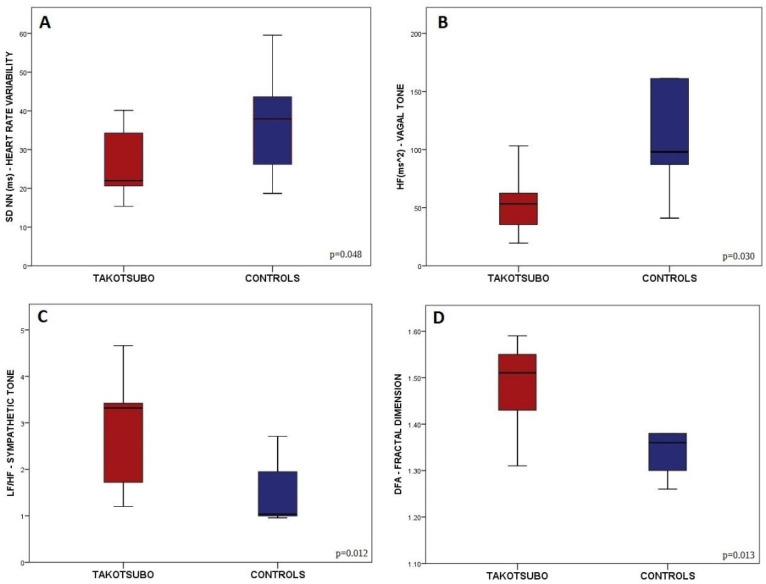
Differences between groups in autonomic status (HRV analysis): SDNN (**A**), HF (**B**), LF/HF (**C**), and fractal dimension (**D**). Data are shown as bar plots with error bars expressed (standard errors).

An overall lower HRV (TS patients SDNN: 26 ± 8; controls SDNN: 38 ± 15; *p* = 0.048; see Figure 2A) was found in TS patients compared to controls, indicating sympathovagal imbalance characterized by an association between hyper-adrenergic tone (TS patients LF/HF: 3.5 ± 2.4; controls LF/HF: 1.6 ± 0.7; *p* = 0.012) and blunted vagal tone (TS patients HF: 65 ± 51; controls HF: 387 ± 273; *p* = 0.030) in TS patients (see Figure 2B,C). Moreover, fractal dimension was higher in TS patients (*p* = 0.013; see Figure 2D), whereas no group differences in HRV entropy were found. Table 4 provides an extended description of the HRV analysis (i.e., time and frequency domain, non-linear analysis) and statistical group comparison.

**Figure 3:**
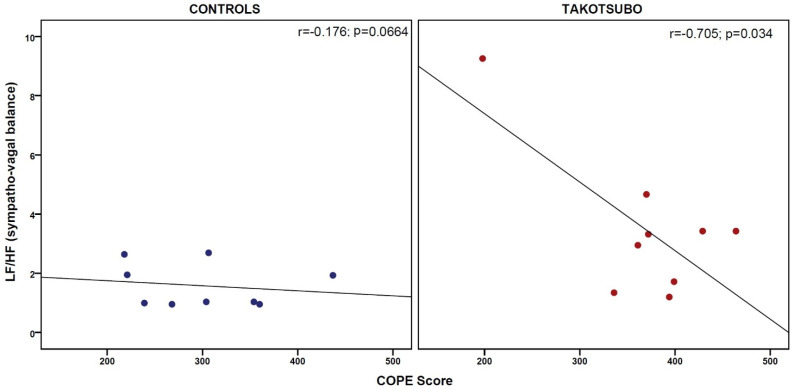
Association between sympathetic tone (LF/HF ratio) and coping orientation (COPE score) in TS patients and healthy controls, separately. In particular, the figure shows a close inverse correlation between hyper-sympathetic tone and reduced coping strategies in TS patients, only.

Correlational analyses between psychological assessment and autonomic status yielded only one significant group difference in the association between sympathetic tone (LF/HF ratio) and coping orientation (COPE score). In particular, only TS patients showed a strong negative correlation between hyper-sympathetic tone and reduced coping strategies (*r* = −0.705; *p* = 0.034; see Figure 3).

## 4. Discussion

**Figure 4:**
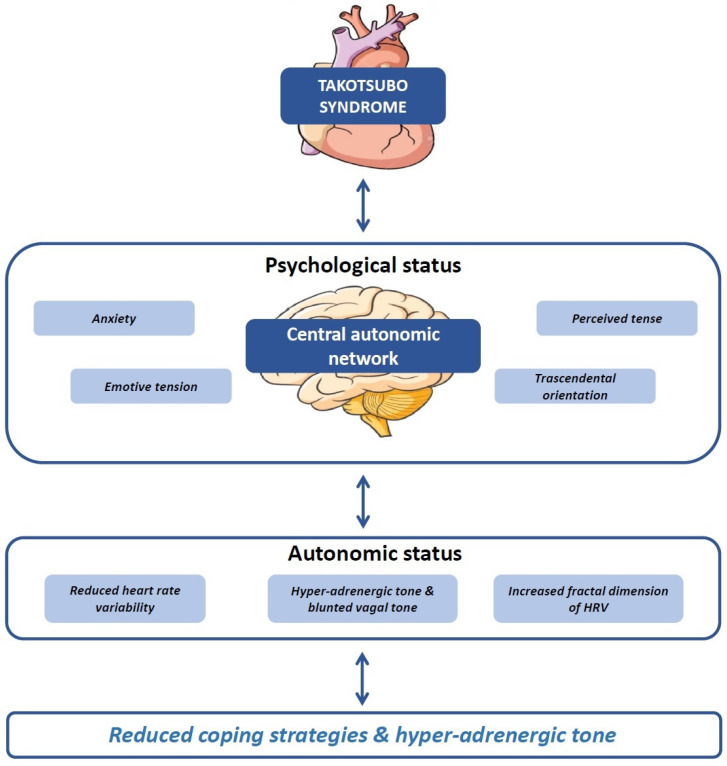
Association between psychological and autonomic status long after the acute phase of TS.

The present study showed the presence of a specific psychological pattern characterizing TS patients long after the acute event, associated with autonomic dysfunction (i.e., sympathovagal imbalance), which is a well-known hallmark of the acute phase of the disease [17]. In particular, higher levels of perceived anxiety with a tendency to feel worry and stress were found in TS patients compared to age- and sex-matched healthy controls. Moreover, TS patients showed a greater transcendental orientation (Cope 5) and higher tension (16 PF-Q4), as well as high apprehension, low vigilance, and emotional stability (aligned with the 16PF global factor of Anxiety, albeit with a difference in the level of vigilance). While not quite statistically significant, lower levels of psychological flexibility and the capacity to be in contact with one’s emotions (AAQ-II), as well as higher levels of perceived depression (BDI-II), were also found in TS patients compared to controls. Moreover, sympathovagal imbalance, characterized by hyper-adrenergic tone, blunted vagal tone, and increased HRV fractal dimension, in addition to higher complexity in the autonomic control of the heart, were found in TS patients. Of interest, in TS patients, an association between hyper-adrenergic tone and overall reduced coping orientation was found, suggesting that the persistence of hyper-sympathetic tone long after the acute phase of TS, reasonably mediated by the central autonomic network, may relate to a reduced ability to cope with stress, trauma, and emotions (see Figure 4).

In recent years, a close link between psychopathology and TS has been widely demonstrated [7,9]. In particular, many studies have observed a high prevalence of anxiety and depression in TS patients [9]. Recently, TS patients in the International Takotsubo Registry were compared with age-matched and sex-matched patients with acute coronary syndrome; TS patients showed a significantly greater prevalence of psychiatric disorders and chronic neurological disease [21]. Anxiety is also commonly associated with TS [7,21], especially when the TS is reported to have been triggered by emotionally stressful events [39]. It may be hypothesized that anxiety traits increase emotional sensitivity to context stimuli, and thus individuals with TS may be easily overwhelmed by stressful life experiences. A recent study [40] on the numerical increase of TS patients during the COVID-19 pandemic provides support for the possible explanatory role played by psychological distress. Moreover, a further study by our research group showed that, relative to ACS patients, TS patients were more frequently affected by psychological disorders [41]. Of note, in our research, anxiety disorders were more frequent in TS patients, while depressive disorders were more frequent in ACS patients [41]. In agreement with these findings, the present results confirm the presence of high levels of perceived anxiety in TS patients. Although the association between TS and anxiety has been widely demonstrated, to date, no study has evaluated the relationship between TS and tension. Factor Q4 of the 16PF is demonstrative of impatience and irritability in response to environmental delays, stressors, and demands [24]. In addition to demonstrating high perceived anxiety levels, the TS patients in the present study also showed high levels of frustration and tension. Only one prior study has assessed the link between TS and the Type-D (i.e., Distressed) personality, characterized by situation-independent negative affectivity and social inhibition [42]. This study revealed a higher prevalence of the Type-D personality among 37 patients with emotionally triggered TS, compared with the same number of patients with myocardial infarction (76% versus 32%, respectively) [42]. Similarly, the present study found higher values of perceived depression (BDI-II) in TS patients.

To the best of our knowledge, only a few studies have evaluated coping strategies and conscious efforts to reduce stress in TS patients. In a retrospective study, Hefner and colleagues [43] compared the responses to a stress-coping questionnaire of 104 healthy participants versus 31 TS patients, finding that TS patients used positive coping strategies, such as de-emphasis and positive self-instruction, less frequently than healthy participants; in contrast, no differences in coping mechanisms were reported between TS patients and patients with myocardial infarction [44]. In the present study, the only difference in coping mechanisms between TS patients and healthy controls referred to a transcendental orientation. Although this coping strategy has never been investigated in TS patients, a higher prevalence of private spiritual activity has been associated with increased cardiovascular risk among post-menopausal women [45]. Religiosity and religious coping have also been identified as variables that may impact an individual’s experience of stress [46].

Religion and spirituality are usually—though not always—beneficial to individuals’ efforts to process trauma; on the other hand, traumatic experiences can lead to a deepening of religiosity or spirituality [47]. The relationship between religious faith and negative life events is undoubtedly complex: while negative life events may strengthen spiritual beliefs, and this may contribute to reducing distress to pre-event levels, contrasting findings have also been produced [48]. The present data, showing a simple association between TS and religious coping long after the acute TS event, cannot clarify whether spirituality preceded or manifested as a response to the stressful event. Of interest, the only anecdotal description linking religion and TS is provided by a case report describing TS triggered by an intense religious discussion [49].

## 5. Conclusions

In conclusion, a close “brain–heart connection” has been long proposed as a critical factor for the development of TS in the acute phase. Psychosocial predisposing factors and acute stressors may play a central role as triggers of an “autonomic cascade” involving several brain autonomic regions, including the limbic system (i.e., insula, hippocampus, amygdala, etc.), the ventromedial prefrontal cortex, and the brainstem [2]. Notably, even after the acute TS event, and despite the full recovery of left ventricular function, autonomic dysfunction and brain structural abnormalities (i.e., insula and amygdala atrophy) may persist, even 1 year later [50]. Nonetheless, it remains unclear whether autonomic dysfunction and psychological alterations represent pre-existing causal elements or consequences of the acute event. In support of the hypothesis that autonomic dysfunction precedes the development of TS, an interesting and recent study showed that patients with subsequent TS had higher baseline (i.e., pre-event) amygdala activity than patients who did not subsequently develop TS [51]. However, in support of central autonomic network involvement, a recent case report showed TS onset during autoimmune limbic encephalitis [52].

The present study, confirming an association between certain psychological patterns and autonomic dysfunction long after the acute TS event, suggests the potential effectiveness of cardiac rehabilitation (CR), including both physical training and psychological management, for TS patients. CR has been shown to be effective at improving both the sympathetic-vagal balance and the psychological profiles of cardiovascular disease patients [1]. Although the benefits of exercise training in TS patients have been hypothesized, CR is underused in TS treatment [53,54]. In fact, there have been no data produced on the effectiveness of CR (in terms of psycho-physical recovery) in TS patients, and a clinical trial is ongoing [55].

The limitations of the current study include the small sample size, the absence of a second control group composed of post–acute coronary syndrome patients, and the lack of pre-event autonomic and psychological data. Thus, although promising, the present results must be considered preliminary, and future studies aimed at replicating the results on a larger sample (i.e., one that is more representative of the TS population) are warmly recommended. These limitations notwithstanding, the present study represents the first simultaneous and integrated evaluation of autonomic function and a wide range of psychological aspects in TS patients long after the acute event. Given the peculiar group differences that emerged, future research should delve further into the possible links between psychological traits and autonomic activity and the mechanisms that may foster such associations in TS patients. 

## Figures and Tables

**Table 1:** Baseline characteristics between TS patients and healthy controls.

	TS Patients	Controls	*p*-Value
Age (years), *M* (*SD*)	69 (7)	72 (7)	0.107
Female, *N* (%)	9 (90)	8 (87)	0.854
BMI, *M* (*SD*)	25 (2)	25 (3)	0.897
Family history of CVD, *N* (%)	2 (20)	1 (11)	0.596
Arterial hypertension, *N* (%)	9 (90)	7 (78)	0.466
Diabetes, *N* (%)	1 (10)	0 (0)	0.330
Dyslipidemia, *N* (%)	7 (70)	5 (55)	0.515
Smoker, *N* (%)	2 (20)	0 (0)	0.156
Anti-hypertensive drugs	9 (90)	7 (78)	0.466

*M* = mean; *SD* = standard deviation; *N* = number; % = percentage; BMI = body mass index; CVD = cardiovascular disease.

**Table 2:** Stressful events in TS patients and in controls (C).

Case	Type of Stressful Event	Description
TS 1	Emotional	Emotional stress during her husband’s funeral
TS 2	Emotional	Emotional stress due to legal issues following her daughter’s death
TS 3	Emotional	Emotional stress (fear/fright) due to her cat having been struck by a car
TS 4	None	No reported stressful event
TS 5	Emotional	Emotional stress while attending an emotionally engaging play
TS 6	None	Chronic stress due to family illness and job loss; no acute stressful event
TS 7	Emotional	Emotional stress during a phone discussion about job problems
TS 8	Emotional	Emotional stress after her son’s university exam
TS 9	Emotional	Emotional stress after her husband’s death, and while awaiting cardiological evaluations for suspected heart disease
TS 10	Emotional	Emotional stress during an argument with her unemployed daughter
C 2	Emotional	Emotional stress due to family illness
C 4	Emotional	Emotional stress due to family illness

**Table 3:** Differences in psychological status between TS patients and healthy controls.

Psychological Evaluation		TS Patients	Controls			
		*M* (*SD*)	*M* (*SD*)	*Kruskal-Wallis H*	*p*-Value	Cohen’s *d*
16PF	Warmth (A)	3.8 (2.0)	4.9 (1.9)	0.65	0.282	0.46
	Reasoning (B)	3.1 (1.8)	3.5 (1.7)	1.49	0.404	0.49
	Emotional Stability (C)	2.9 (1.6)	5.1 (3.3)	1.62	0.180	0.82
	Dominance (E)	4.3 (2.3)	3.3 (2.2)	0.49	0.556	0.38
	Liveliness (F)	5.5 (2.1)	4.0 (2.9)	2.30	0.265	0.87
	Rule-Consciousness (G)	5.8 (1.7)	4.3 (2.0)	2.20	0.106	0.77
	Social Boldness (H)	3.2 (1.1)	2.1 (1.4)	2.43	0.086	0.77
	Sensitivity (I)	7.6 (1.7)	6.8 (1.8)	1.01	0.291	0.44
	Vigilance (L)	5.2 (2.5)	5.0 (2.4)	0.05	0.967	0.14
	Abstractedness (M)	5.3 (2.1)	4.8 (2.1)	0.34	0.772	0.37
	Privateness (N)	4.9 (2.6)	4.7 (3.1)	0.01	0.835	0.13
	Apprehension (O)	7.5 (1.4)	6.2 (1.7)	1.98	0.102	0.71
	Openness to Change (Q1)	3.2 (1.5)	3.4 (1.4)	0.05	0.802	0.14
	Self-Reliance (Q2)	2.6 (1.7)	2.9 (2.2)	0.10	0.833	0.13
	Perfectionism (Q3)	2.3 (0.9)	4.1 (3.0)	0.99	0.338	0.80
	Tension (Q4)	8.4 (0.6)	7.0 (1.5)	6.04	0.028 *	1.34
	O Q4	15.9 (1.6)	13.2 (2.8)	4.24	0.027 *	1.17
	O Q3	9.5 (1.5)	10.3 (3.9)	0.16	0.588	0.26
	O+ Q+ C−	12.6 (2.6)	8.1 (5.8)	2.18	0.048 *	1.03
	O+ Q4+ C− L	7.3 (3.0)	2.9 (5.4)	4.33	0.047 *	1.02
	MD (16 PF-C)	2.7 (0.6)	3.0 (2.0)	0.01	0.866	0.15
AAQ-II	Psychological Flexibility	42.7 (9.1)	50.6 (11.4)	2.68	0.072	0.73
STAI	STAI-Y1—State Anxiety	2.7 (2.7)	0.8 (2.1)	2.39	0.121	0.80
	STAI-Y2—Trait Anxiety	2.3 (1.5)	0.7 (1.5)	4.31	0.041 *	1.06
BDI-II	Depressive Symptoms	74 (24)	49 (26)	2.18	0.071	0.87
COPE	COPE 1	34 (8)	29 (5)	2.55	0.151	0.81
	COPE 2	32.7 (8)	32 (7)	0.01	0.967	0.13
	COPE 3	34.7 (7.6)	35.1 (6.5)	0.02	1.000	0.01
	COPE 4	32.8 (4.2)	34.3 (5.8)	1.05	0.345	0.33
	COPE 5	12.1 (4.9)	6.8 (2.6)	7.20	0.002 *	1.78

*M* = mean; *SD* = standard deviation; 16PF = 16 Personality Factors-C Form; AAQ-II = Acceptance and Action Questionnaire-II; COPE = Coping Orientations to Problems Experienced; BDI-II = Beck Depression Inventory-II; STAI = State Trait Anxiety Inventory; * = *p*-value below 0.05.

**Table 4:** Differences in autonomic status between TS patients and healthy controls.

Autonomic Evaluation		TS Patients	Controls			
		*M* (*SD*)	*M* (*SD*)	*Kruskal–Wallis H*	*p*-Value	Cohen’s *d*
Time domain	SDNN (ms)	26 (8)	38 (15)	3.49	0.048 *	1.02
	pNN50 (%)	0 (0)	1 (1)	8.60	0.003 *	1.05
	RMSSD (ms)	17 (6)	44 (29)	5.91	0.015 *	1.29
Frequency domain	VLF (ms^2^)	206 (170)	245 (194)	0.16	0.691	0.21
	LF (ms^2^)	11,744 (3472)	434 (321)	0.70	0.401	0.46
	HF (ms^2^)	65 (51)	387 (273)	4.69	0.030 *	0.81
	LF/HF (ms^2^)	3.5 (2.4)	1.6 (0.7)	6.35	0.012 *	1.07
Non-linear analysis	SamEn	2.480 (2.350)	2.491 (2.106)	0.34	0.560	0.01
	FD	1.48 (0.09)	1.35 (0.09)	6.15	0.013 *	1.32

*M* = mean; *SD* = standard deviation; SDNN = standard deviation of NN intervals; pNN50 = percentage of successive RR intervals that differ by more than 50 ms; RMSSD = root mean square of successive RR interval differences; VLF = very-low-frequency band (0.0033–0.04 Hz); LF = low-frequency band (0.04–0.15 Hz); HF = high-frequency band (0.15–0.4 Hz); LF/HF = ratio of LF to HF power; SampEn = sample entropy; FD = fractal dimension; * = *p*-value below 0.05.

## Data Availability

The data presented in this study are available only upon reasonable request to the corresponding author due to privacy restrictions.
